# Engineering Pareidolia: Mental Imagery, Perceptual Scaffolding, and Visual Creativity

**DOI:** 10.3390/brainsci16030321

**Published:** 2026-03-17

**Authors:** Alexis Demas

**Affiliations:** ARGOS (Art, Researchs and Gestures Observed Scientificaly) Neurosciences and Arts, 229 Rue Saint-Honoré, 75001 Paris, France; alexis.demas@yahoo.fr or dr.alexisdemas@gmail.com

**Keywords:** pareidolia, mental imagery, perceptual scaffolding, predictive processing, face perception, creative perception, divergent perception, neuroaesthetics, Lewy body disease

## Abstract

**Highlights:**

**What are the main findings?**

- Engineered pareidolia can be framed as a form of externally scaffolded mental imagery, in which minimal stimulus constraints elicit stable, template-based completion.
- Art-historical exemplars (Arcimboldo; Dürer; Leonardo) illustrate distinct “design regimes” through which sparse cues can reliably trigger face-like completion via top-down inference.

**What are the implications of the main findings?**

- Engineered pareidolia may offer tractable paradigms to quantify creative perception (detection thresholds, robustness to perturbation, inter-observer reproducibility) and relate it to neural signatures.
- The framework could help bridge neuroaesthetics with clinically quantifiable pareidolia in Lewy body disease, informing models of altered precision/priors under uncertainty.

**Abstract:**

Pareidolia is often framed as a viewer-side illusion: a tendency to perceive meaningful forms—especially faces—in ambiguous inputs. This Concept Paper argues that pareidolia can also be deliberately *engineered* and therefore provides a tractable entry point into the neurophysiology of visual creativity. We propose a unifying construct in which engineered pareidolia functions as *externally scaffolded mental imagery*: minimal visual constraints recruit internally generated templates and top-down inference while remaining anchored to sensory input. To strengthen theoretical rigor, we define necessary and sufficient features that distinguish this construct from adjacent accounts (scaffolded cognition; perceptual scaffolding; bistable perception). Using Arcimboldo’s composite portraits and Dürer’s embedded face in *View of the Arco Valley*, plus a canonical Renaissance example (Leonardo’s *Bacchus/Saint John the Baptist*), we outline distinct “design regimes” that modulate cue validity, attentional release, and interpretive switching. We then connect engineered pareidolia to creativity research by linking pareidolia design and detection to measurable constructs in divergent/creative perception, including but not limited to Torrance-style domains, and we propose feasible behavioral and neurophysiological paradigms that control for artistic skill and clinical status. Finally, we distinguish benign pareidolia from hallucination, discuss clinical resonance in dementia with Lewy bodies where pareidolia can be quantified, and outline an empirically testable research program that reframes pareidolia as a bridge between imagination, perception, and creativity.

## 1. Introduction

Mental imagery and visual perception share representational resources while remaining dissociable in how strongly they are constrained by sensory input and controlled by attention [1,2,3,4,5]. Individual differences—from aphantasia to unusually vivid imagery—support the idea that imagery–perception coupling is a dimension of brain function rather than a binary state [5,6].

Pareidolia sits at this interface. It is the experience of perceiving meaningful forms (often faces) in inputs that underspecify those forms. Face pareidolia can recruit face-selective processing rapidly, consistent with template-driven inference under ambiguity [7]. Clinically, pareidolia is not merely anecdotal: it can be evoked and quantified in dementia with Lewy bodies (DLB), and relates to vulnerability to complex visual illusions/hallucinations [8,9,10,11]. Neurocomputational accounts propose that such phenomena reflect altered precision-weighting and increased reliance on prior knowledge when sensory evidence is uncertain [12,13,14].

This paper advances a specific thesis: **pareidolia can be deliberately engineered**, and engineered pareidolia is not just “illusion in the viewer,” but a **designed interface** between stimulus constraints and internally generated imagery-like completion. The goal is not to claim established facts without data, but to (i) define an operational construct, (ii) ground it in empirical constraints from perception and face-processing research, and (iii) map it to creativity research in a way that yields testable predictions.

## 2. Defining “Externally Scaffolded Mental Imagery” (ESMI)

The phrase “externally scaffolded” has multiple neighbors in the literature (extended cognition; scaffolded cognition; perceptual scaffolding) [15,16,17]. To avoid conceptual vagueness, the construct proposed here is defined operationally.

### 2.1. Operational Definition (Necessary and Sufficient Features)

**Externally scaffolded mental imagery (ESMI)** is defined as a perceptual–imagery hybrid state with:

**(N1) Minimal external constraints:** The stimulus provides *insufficient* bottom-up information to uniquely specify a category (e.g., a face) under purely feedforward decoding.

**(N2) Template recruitment:** A category template (e.g., face schema) is recruited to supply missing structure (completion).

**(N3) Stimulus anchoring:** The resulting experience remains *locked* to external spatial constraints (it is not free-floating imagery).

**(N4) Reproducibility across observers/conditions:** The engineered constraints yield *non-trivial* inter-observer agreement and robustness across controlled transformations (blur, inversion, contrast shifts).

**(N5) Measurability:** The state yields quantifiable signatures (behavioral thresholds; face-selective neural markers; attention effects).

These criteria distinguish ESMI from:

- **Pure imagery** (lacks N3 anchoring);
- **Ordinary perception** (violates N1 because sensory evidence is sufficient);
- **Generic bistability** (may lack N2 template specificity);
- **Broad “scaffolded cognition”** accounts (too general unless tied to N1–N5) [15,16].

### 2.2. Terminology Standardization

To reduce interchangeability:

- **Template** = stored category model (e.g., face schema).
- **Completion** = top-down filling-in that supplies missing structure.
- **Attractor** = a stable interpretive state toward which perception converges under ambiguity (dynamical systems framing).

These are used consistently below.

## 3. Why Pareidolia, Specifically, as a Bridge to Creativity?

Many ambiguity-based phenomena exist (Mooney faces, closure, binocular rivalry, Necker cube). Yet pareidolia—especially face pareidolia—has three advantages as a creativity bridge:

- **Category privilege and sensitivity:** Faces are socially salient, processed by specialized/distributed systems, and can be triggered by sparse configurational cues [18,19].
- **Parametric controllability:** Pareidolia can be titrated by image statistics, orientation, spectral power, and cue validity, allowing threshold curves rather than binary reports [20,21].
- **Clinical tractability:** In DLB, pareidolia can be elicited and quantified with structured tests, linking basic inference mechanisms to clinical outcomes [8,9,10,11].

Crucially, recent empirical work has begun connecting pareidolia to **creative perception**: creative individuals may detect recognizable forms in ambiguous fractal patterns more readily and across broader stimulus ranges [22]. And “divergent pareidolia” production tasks have been proposed as a novel way to measure creative cognition [23]. These findings make pareidolia a plausible *operational handle* rather than a purely metaphorical bridge.

## 4. Predictive Processing: Which Version Is Assumed?

When ambiguity rises, perceptual inference depends on how the brain weights sensory evidence versus priors. Here, “predictive processing” is used in the **precision-weighted predictive coding** sense: perception reflects hierarchical Bayesian inference where prediction errors are weighted by their estimated precision, and action/attention can be conceptualized as precision control (active inference-compatible framing) [13,14]. Under this view, pareidolia is not “mistake,” but a regime in which priors or precision estimates dominate under uncertainty.

## 5. Engineered Pareidolia as “Predictive Engineering”

If pareidolia can be engineered, the artwork functions as a designed perturbation of inference. The creator is not merely producing an image, but shaping a viewer’s interpretive trajectory by controlling:

- **Cue validity** (what minimal features are sufficient to recruit a template);
- **Attentional gating** (what must be ignored or disengaged for the latent percept to emerge);
- **Attractor competition** (how strongly a dominant reading suppresses a latent reading).

This framing does not require assuming artists explicitly formalized predictive coding; it only claims that artistic practice can instantiate its functional consequences.

## 6. Three Art-Historical Regimes of Engineered Pareidolia

### 6.1. Arcimboldo: Hierarchical Composite Faces

**Figure 1:**
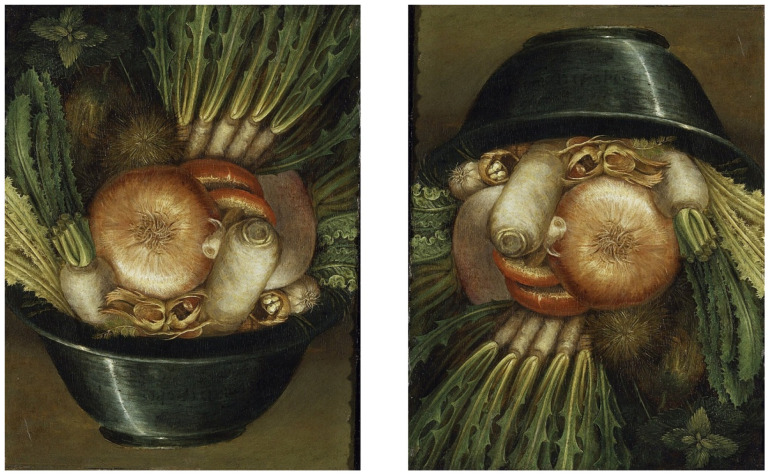
Arcimboldo and hierarchical composite faces (engineered global configuration). Example of Arcimboldo-style composite portrait logic: a global face configuration emerges from non-face objects, forcing competition between a face template at the global level and object identity at the local level. This regime predicts measurable face-selective signatures when the global configuration is preserved, and reduction when inversion disrupts holistic processing [25]. Image: public-domain reproduction (source credited in final layout).

Arcimboldo’s composite portraits exemplify a regime where global configuration recruits a face template while local parts remain non-face objects (Figure 1). This forces a negotiation between global and local processing [24]. Importantly, electrophysiological evidence constrains the theory: Arcimboldo-like portraits elicit the N170 component in a manner closer to faces than objects when the global configuration is face-like, and inversion reduces this effect—consistent with early holistic face-like processing rather than mere post-perceptual labeling [25]. This does not prove imagery, but it supports N2 (template recruitment) and N5 (measurable markers) under engineered constraints.

### 6.2. Dürer: Embedded Faces and Attentional Release

**Figure 2:**
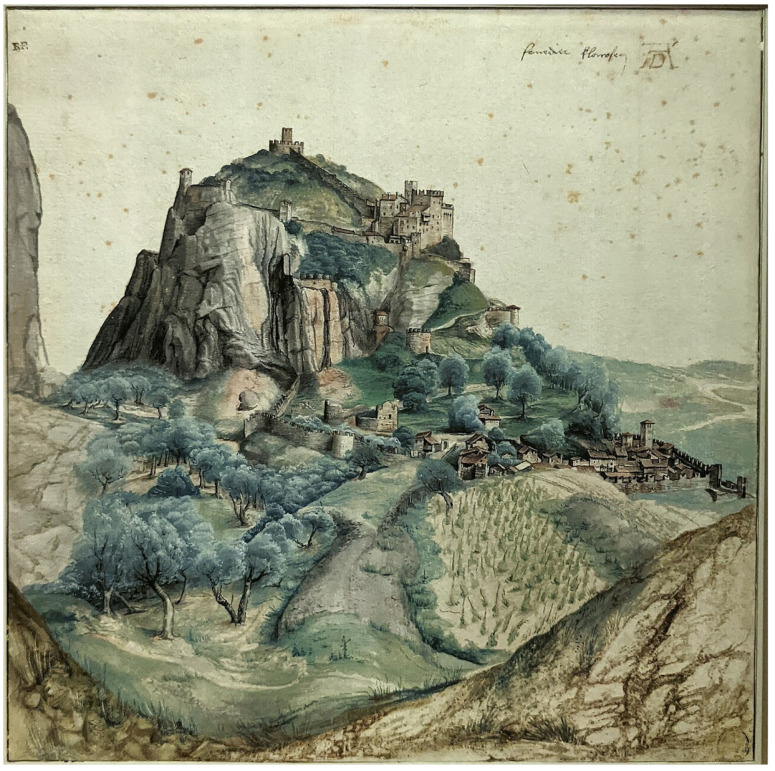
Dürer and embedded pareidolia (attentional release and attractor switching). *View of the Arco Valley* (c. 1495). A latent profile can be discerned in the cliff structure (annotation/outline recommended in the figure panel). The proposed “two-stage interpretation” is framed as a testable hypothesis: a dominant landscape attractor organizes perception until attention/instructions/viewing distance enable a switch to a face attractor, consistent with attention-biased switching in ambiguous/bistable perception [26,27]. Image: public-domain reproduction (source credited).

Dürer’s *View of the Arco Valley* illustrates a different regime: a latent face-like profile embedded in a naturalistic landscape (Figure 2). The “two-stage interpretation” proposed here is framed as a testable hypothesis: an initial dominant landscape attractor organizes gaze and suppresses alternative readings; a second attractor (face) emerges when attention shifts, viewing distance changes, or instructions bias interpretation. This is consistent with broader evidence that attention can bias perceptual switching in bistable perception and that ambiguous stimuli can yield competing stable interpretations [26,27]. In this regime, engineered pareidolia is less about explicit compositional assembly and more about **designing the conditions for perceptual release**.

### 6.3. Leonardo: Engineered Pareidolia as “Cryptic Dialogue” Under Controlled Viewing

**Figure 3:**
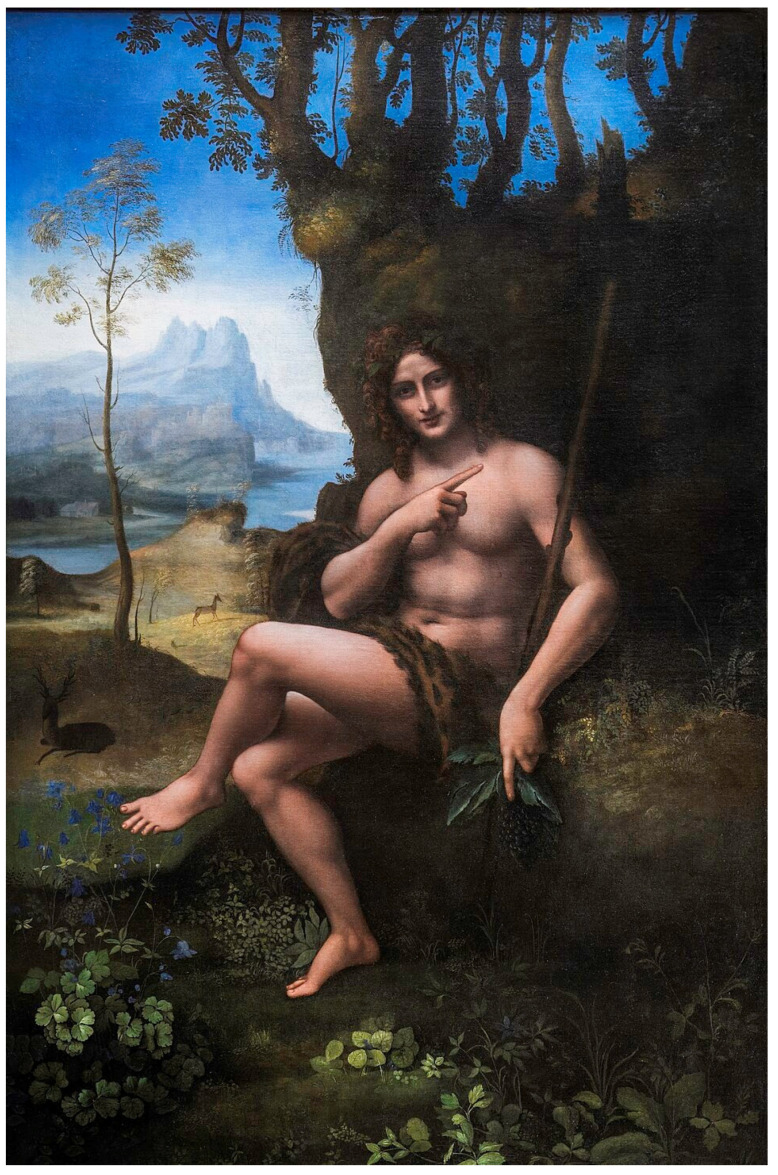
Leonardo and gated pareidolia (viewing-condition dependence). Example of engineered pareidolic faces embedded within the background of Leonardo’s *Bacchus/Saint John the Baptist*. The panel should include (A) the original crop; (B) a contrast/illumination-adjusted version; (C) a minimal annotation indicating the facial configuration cues (e.g., eye-like dark spots, nasal ridge, mouth-like contour). The intent is to make the figure self-explanatory without requiring consultation of external sources, while illustrating a regime where detection is contingent on attentional disengagement from the main attractor and on viewing conditions [28].

A canonical Renaissance case illustrates a third regime: pareidolic faces embedded in the background of Leonardo’s *Bacchus/Saint John the Baptist*, reported as requiring specific viewing conditions (contrast/illumination changes; attentional disengagement from the central figure) for reliable detection (Figure 3) [28]. This example is valuable because it formalizes a practical claim: the artist may design not only the latent form but also the **gating mechanism**—a controlled pathway by which a viewer moves from one attractor to another.

## 7. Creativity Bridge: From Divergent Thinking to Divergent Perception

Classic creativity assessment emphasizes divergent thinking (fluency, originality, elaboration) and resistance to premature closure [29]. Yet pareidolia offers a complementary axis: **divergent perception**, the ability to detect or generate meaningful structure under ambiguity [22,23].

Engineered pareidolia maps naturally onto modern creativity models that emphasize the interplay between associative generation and executive selection/control [30,31]. The creator explores candidate mappings between ambiguous structure and meaningful templates, then selects a mapping that remains robust across transformations and across observers (N4). In this view, “resistance to premature closure” becomes a perceptual discipline: the creator must hold ambiguity long enough to test multiple completions before converging on a stable engineered design.

Drawing and image-making research strengthens the plausibility of this bridge: drawing is increasingly framed as a versatile cognitive tool that externalizes internal models and makes mental content publicly inspectable, supported by top-down attentional control and expertise effects [17].

## 8. Testable Paradigms and Feasibility Details

A Concept Paper is strongest when it specifies a program that can realistically be executed.

### 8.1. Pareidolia Creation Task (Designer-Side)

**Participants:** trained visual artists vs. matched controls (age/education), plus an optional DLB group for clinical comparison.

**Task:** participants modify ambiguous textures/noise images to maximize face detection by naïve raters.

**Outcomes:** naïve detection rate, reaction times, confidence, robustness across transformations (blur/inversion/contrast), inter-rater agreement.

**Controls for artistic skill:** baseline drawing/visual editing task; independent ratings of technical proficiency; within-subject counterbalancing of tool familiarity; inclusion of “non-face target” control conditions.

This directly operationalizes “predictive engineering”: the creator aims to maximize N4 robustness and N5 measurability.

### 8.2. Pareidolia Detection Threshold Task (Viewer-Side)

**Stimuli:** parametric Arcimboldo-like composites with graded disruption of global configuration; Dürer-like embedded profiles with graded contour cues; controlled image statistics.

**Outcomes:** psychometric detection curves; sensitivity to orientation and spectral power; relation to imagery vividness measures and creative perception indices [21,22].

**Neurophysiology:** EEG N170/FPVS for face-selective signatures; attention manipulation (instructional set) to test gating.

### 8.3. Linking to Clinical Pareidolia (DLB)

**Rationale:** DLB pareidolia tests provide a structured, clinically meaningful measure that can be linked to altered inference/precision [8,9,10,11,12].

**Design:** compare benign pareidolia in healthy observers with clinical pareidolia in DLB using matched tasks; examine whether differences are explained by altered precision weighting, attentional control, or sensory reliability.

## 9. Cultural Functions (Concrete Examples)

Reviewer feedback rightly notes that “cryptic symbolism” must be grounded. Engineered pareidolia can serve at least two concrete cultural functions:

**Virtuoso puzzles and courtly games:** Arcimboldo operated in court environments where riddles, reversals, and cognitive games were valued. Composite portraits could function as elite perceptual challenges, displaying control over a viewer’s inference while remaining deniable as playful invention. This is exemplified by *Vertumnus* (Rudolf II), where the composite face also carries political-allegorical messaging about abundance, order, and sovereign mastery [32,33].

**Layered communication via attentional gating:** In works where the latent percept emerges only under altered viewing or disengaged attention (Figure 2 and Figure 3), the artwork can support a “two-channel” message: a primary, socially acceptable reading and a secondary reading accessible to those who “know how to look.” This does not require asserting a single hidden agenda; it only requires acknowledging that engineered pareidolia is a plausible mechanism for layered meaning because it exploits how attention and templates govern perception.

## 10. Clinical Resonance: Benign Completion vs. Hallucination

A key distinction is between **benign pareidolia** (a context-sensitive completion that observers can typically revise) and **hallucination/clinical intrusion** (percepts that can become obligatory, distressing, and less corrigible). In DLB, pareidolia can be reliably elicited and correlates with vulnerability to complex visual phenomena [8,9,10,11,12]. Predictive accounts interpret this as altered weighting of priors or reduced sensory precision under uncertainty [12,13,14].

The conceptual reciprocity is important: the same inferential architecture that enables creativity and meaning-making can, under altered precision control, generate intrusive phenomena. A mature science of creative perception should therefore study parameters that shift completion from chosen to imposed.

## 11. Conclusions and Outlook

This Concept Paper argues that pareidolia is not only a viewer-side illusion but can be **engineered** as a reproducible perceptual outcome. We define engineered pareidolia as **externally scaffolded mental imagery** (ESMI) using operational criteria that distinguish it from adjacent constructs. Arcimboldo, Dürer, and Leonardo exemplify distinct design regimes that manipulate cue validity, attentional release, and attractor competition. Because face pareidolia is measurable, parametric, and clinically tractable, it may provide a rare bridge between neuroaesthetics, creativity science, and clinical neuropsychology.

**Outlook:** The next step is not broader theorizing but targeted experiments: creator-side pareidolia design tasks with naïve detection benchmarking; viewer-side threshold paradigms with EEG/FPVS markers; and clinical comparisons in DLB using matched stimuli. If successful, engineered pareidolia could become a quantitative platform for studying creative perception as precision-controlled completion under constraint—linking culture, cognition, and disease without collapsing any domain into the other.

## Data Availability

No new data were created or analyzed in this study.
